# Management of Ankle Sprains in Urgent Care: Adherence to Evidence-Based Guidelines

**DOI:** 10.7759/cureus.106852

**Published:** 2026-04-11

**Authors:** Maram M Alharbi, Musaad Al Musaad

**Affiliations:** 1 Department of Family Medicine, Prince Sultan Military Medical City, Riyadh, SAU

**Keywords:** acute ankle sprain, ottawa ankle rules, physician guideline adherence, rehabilitation protocol, urgent care clinic

## Abstract

Background

Ankle injuries are among the most common reasons for urgent care visits. Evidence-based guidelines, particularly the Ottawa Ankle Rules (OAR), aim to standardize assessment and reduce unnecessary imaging. Despite strong evidence supporting their diagnostic accuracy, clinicians' adherence remains variable. This variability can lead to suboptimal patient outcomes, such as chronic joint instability and increased healthcare costs. This study assesses physicians' compliance with OAR and related management recommendations and identifies barriers that hinder appropriate guideline use.

Objectives

The primary objective is to measure the prevalence of adherence to evidence-based guidelines, specifically the OAR, among urgent care physicians. Secondary objectives include evaluating physicians' self-reported familiarity with these guidelines, identifying associated factors affecting compliance, and exploring perceived barriers to implementation.

Methods

A cross-sectional analytical survey was distributed electronically to physicians working in urgent care and emergency services. A total of 74 participants completed the questionnaire. The survey addressed physicians' use of OAR, rehabilitation advice, perceived barriers, and training background.

Results

While only 41.9% routinely applied structured rehabilitation protocols, 70.3% of physicians reported using the OAR. Most respondents (83.8%) provided patient education regarding prevention and rehab. However, 68.9% indicated a need for further training. The most commonly cited barriers were time constraints (32.4%), inadequate guideline training (31.1%), insufficient resources (14.9%), and patient non-adherence (19.4%). Additionally, 87.7% had not attended recent workshops, though 85.1% expressed willingness to receive further education.

Conclusion

The study reveals inconsistencies in adhering to ankle injury guidelines in urgent care. Enhancing clinician training and implementing standardized protocols can reduce diagnostic errors and unnecessary imaging. Ongoing education and quality improvement initiatives are essential for providing better care for ankle injuries.

## Introduction

Ankle sprains are a significant concern among musculoskeletal injuries and a common issue managed in urgent care and emergency departments. These injuries represent a substantial proportion of lower leg injuries and annual healthcare presentations [1-3]. Even minor injuries can lead to chronic sequelae, including disability, persistent pain, and restricted range of motion [4-6]. Suboptimal management may further exacerbate these issues, potentially leading to chronic ankle instability and recurrent sprains [4-7]. Therefore, accurate diagnosis and evidence-based management are crucial for preventing long-term functional impairment [1,4-7].

Clinical guidelines, such as the Ottawa Ankle Rules (OAR), help guide imaging decisions and reduce unnecessary radiation [8,9]. Studies show OAR are highly sensitive for ruling out fractures in acute ankle injuries [8,9]. However, OAR are not always used, and many clinicians, although familiar with them, do not consistently follow them [3,10].

Global consensus highlights the effectiveness of interventions for ankle sprains, supporting early mobilization, functional bracing, and structured rehabilitation for optimal recovery [5-7,10]. Compared to prolonged immobilization, functional treatments consistently yield better results, including faster recovery, earlier return to activities, and a lower risk of recurrence [1,5,11]. However, implementing these proven techniques is often hindered by gaps in guidelines, which frequently overlook key facilitators, barriers, resource needs, and differences in the education and training of healthcare professionals [2,6,12]. Given this, it is crucial to evaluate how effectively urgent care physicians adhere to these protocols. This study aims to evaluate the use of evidence-based guidelines in urgent care by examining clinicians' awareness, perceived barriers, and current practices, with the ultimate goal of enhancing clinical decision-making, improving patient outcomes, and achieving a measurable reduction in unnecessary imaging. By targeting this specific outcome, the study aims to provide actionable insights that drive tangible changes in practice, ultimately contributing to more efficient, cost-effective patient care.

## Materials and methods

The study employed a cross-sectional survey design to assess physicians’ compliance with evidence-based practices for the management of ankle sprains in emergency and urgent care settings. The target population consisted of physicians working in emergency and urgent care departments at Prince Sultan Military Medical City (PSMMC) in Riyadh. Data were collected between February 2025 and October 2025. Inclusion criteria comprised residents, registrars, and consultants with specialties in family medicine and emergency medicine. Exclusion criteria included physicians from other specialties, medical students, and incomplete responses. Data were collected using a structured questionnaire developed by the author to assess demographic characteristics, awareness of clinical guidelines (including the OAR), current management practices, and perceived barriers to guideline adherence. The questionnaire was distributed electronically via email and WhatsApp groups (Meta Platforms, Inc., Menlo Park, CA, USA), with technical settings enabled to prevent duplicate responses (one response per unique user), as well as through a digital link provided to physicians during in-person clinical visits.

Data analysis used descriptive statistics (frequencies and percentages) to summarize demographic and practice-related variables. Adherence was operationally defined as the self-reported frequency of OAR application in clinical practice. Due to the ordinal nature of the data (Likert-scale variables) and the small sample size in some subgroups, inferential analysis was conducted using non-parametric tests. The Mann-Whitney U test and Kruskal-Wallis test were used to examine associations between physicians’ characteristics (such as gender, specialty, and years of experience) and adherence to clinical guidelines. Statistical significance was set at p < 0.05.

Ethical approval was obtained from the Institutional Review Board (IRB) of PSMMC (Approval No. E-2499). Informed consent was obtained from all participants, and confidentiality and voluntary participation were ensured. The questionnaire content validity was confirmed through expert review by two family medicine consultants and one emergency medicine consultant. A pilot study involving five physicians was conducted to refine the questionnaire (specifically to resolve ambiguous phrasing and layout issues), and pilot responses were excluded from the final analysis. The full questionnaire is provided in the Appendix.

## Results

Demographic characteristics

A total of 74 physicians were included in the analysis (Table 1). Most participants were aged 30-39 years (n = 40, 54.1%), followed by those under 30 years of age (n = 27, 36.5%). Smaller proportions were aged 40-49 years (n = 3, 4.1%) or 50 years and older (n = 4, 5.4%).

**Table 1:** Demographic and practice characteristics of participating physicians (N = 74) PSMMC: Prince Sultan Military Medical City

Variable	Category	Frequency (n)	Percent (%)
Age group	Under 30 years	27	36.5
	30-39 years	40	54.1
	40-49 years	3	4.1
	50 years or older	4	5.4
Gender	Male	41	55.4
	Female	33	44.6
Clinical experience	<5 years	39	52.7
	5-10 years	27	36.5
	11-20 years	4	5.4
	>20 years	4	5.4
Specialty	Family medicine	63	85.1
	Emergency medicine	9	12.2
	Orthopedics	1	1.4
	Other	1	1.4
Practice setting	Primary health center	46	62.2
	General hospital	27	36.5
	PSMMC	1	1.4

Male physicians constituted 55.4% of the sample (n = 41), while female physicians accounted for 44.6% (n = 33). Regarding clinical experience, more than half of the participants had less than five years of experience (n = 39, 52.7%), followed by those with 5-10 years of experience (n = 27, 36.5%). Physicians with 11-20 years and more than 20 years of experience each represented 5.4% of the sample (n = 4).

Family medicine physicians comprised the majority of respondents (n = 63, 85.1%), followed by emergency medicine physicians (n = 9, 12.2%). Most participants were affiliated with primary healthcare centers (n = 46, 62.2%), while 36.5% were working in general hospitals (n = 27).

Knowledge and familiarity with guidelines

**Figure 1:**
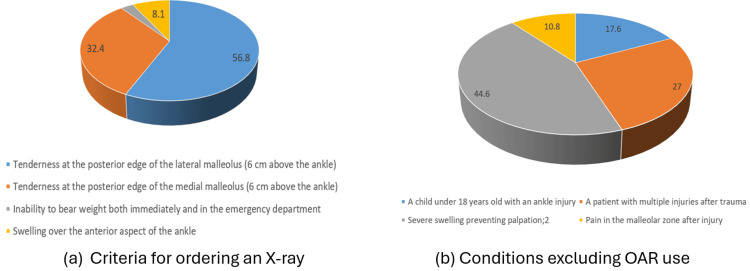
Physicians' knowledge of the Ottawa Ankle Rules (OAR) (a) Criteria identified by participants for ordering an ankle X-ray based on OAR. (b) Conditions identified that exclude the use of OAR.

While nearly all participating physicians reported familiarity with the OAR, with 71 physicians (95.9%) indicating awareness (Table 2), this self-reported familiarity contrasted with their detailed knowledge of the specific criteria. When identifying criteria for ordering ankle radiographs based on the OAR (Figure 1a), only 56.8% (n = 42) correctly selected tenderness at the posterior edge of the lateral malleolus as a key criterion, followed by tenderness at the posterior edge of the medial malleolus (n = 24, 32.4%). This discrepancy suggests a notable gap between perceived familiarity and accurate clinical recall among the participants. Regarding exclusions to OAR application (Figure 1b), severe swelling preventing adequate palpation was the most commonly reported exclusion factor (n = 33, 44.6%), followed by the presence of multiple injuries after trauma (n = 20, 27%).

**Table 2:** Physicians’ knowledge, practice patterns, and adherence to guidelines AAFP: American Academy of Family Physicians; NICE: National Institute for Health and Care Excellence

Item	Response	Frequency (n)	Percent (%)
Familiarity with Ottawa Ankle Rules (OAR)	Yes	71	95.9
	No	3	4.1
Guidelines used for management	AAFP	46	62.2
	NICE	26	35.1
	Other	2	2.7
Awareness of early mobilization benefits	Yes	60	81.1
	No	14	18.9
Educates patients on prevention	Yes	62	83.8
	No	12	16.2
Use of structured rehabilitation protocols	Yes	31	41.9
	No	43	58.1

Clinical practice patterns and adherence

**Figure 2:**
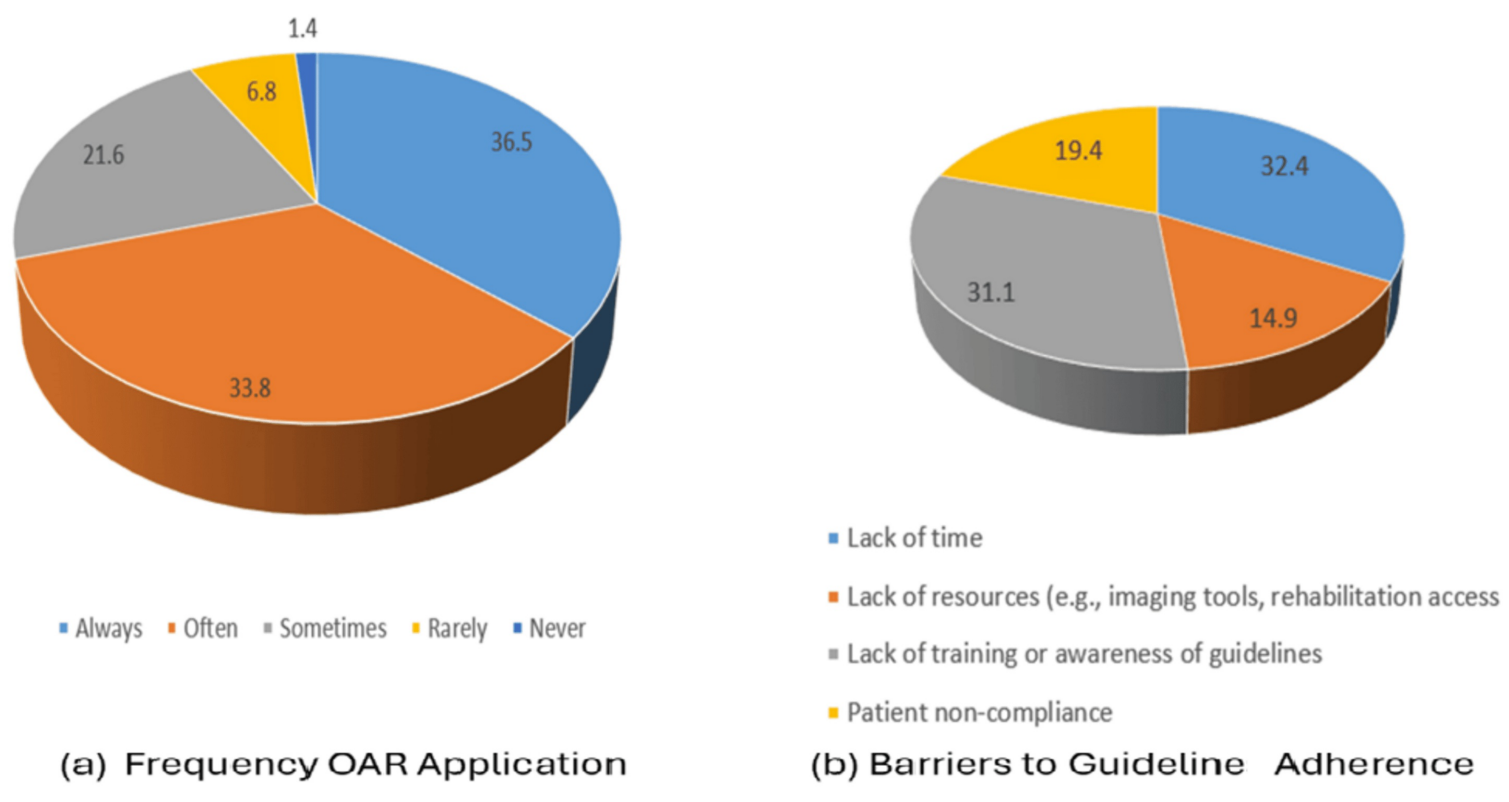
Adherence and barriers (a) Frequency of applying OAR. (b) Reported barriers to adherence. Data are presented as frequency (N) and percentage (%). OAR: Ottawa Ankle Rules

Most physicians (n = 60, 81.1%) reported awareness of the benefits of early mobilization and rehabilitation for ankle sprains (Table 2). In terms of OAR application in daily practice, 36.5% of physicians reported always applying the rules (n = 27), 33.8% reported using them often (n = 25), and 21.6% reported occasional use (n = 16) (Figure 2a).

Statistical analysis demonstrated no significant association between the frequency of OAR application and years of clinical experience (Kruskal-Wallis test: H = 1.65, p = 0.648). Similarly, no significant difference in OAR usage frequency was observed between male and female physicians (Mann-Whitney U = 640.0, p = 0.680) (Table 3). However, these non-significant findings should be interpreted with caution. Given the very small sample sizes in the higher-experience categories (n = 4 in both the 11-20 and >20 years groups), the study was likely underpowered to detect a true difference even if one exists, presenting a risk of a Type II error.

**Table 3:** Statistical analysis of factors associated with the frequency of Ottawa Ankle Rules (OAR) application Statistical significance was set at p < 0.05.

Variable	Category	Frequency (n)	Percent (%)	Statistical test	Test value	p-value
Gender	Male	41	55.4	Mann-Whitney U	U = 640.0	0.680
	Female	33	44.6			
Years of experience	<5 years	39	52.7	Kruskal-Wallis	H = 1.65	0.648
	5-10 years	27	36.5			
	11-20 years	4	5.4			
	>20 years	4	5.4			

**Figure 3:**
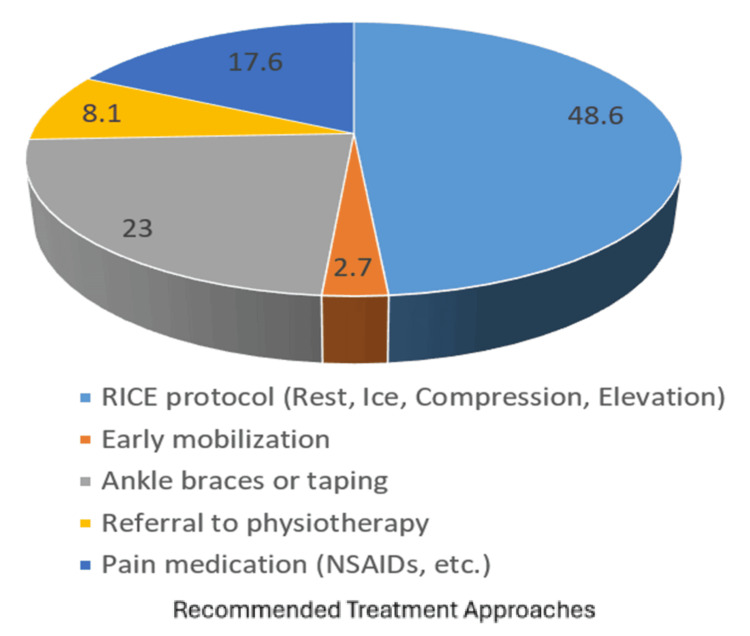
Commonly recommended treatment approaches for ankle sprains Data are presented as frequency (N) and percentage (%). NSAIDs: non-steroidal anti-inflammatory drugs

Regarding treatment approaches (Figure 3), the RICE (rest, ice, compression, and elevation) protocol was the most commonly recommended strategy (n = 36, 48.6%), followed by ankle bracing or taping (n = 17, 23%). Structured rehabilitation protocols were reported by 41.9% of physicians (n = 31), while 58.1% (n = 43) indicated they did not routinely use standardized rehabilitation programs.

Barriers and educational needs

As illustrated in Figure 2b, the most frequently reported barriers to guideline adherence were lack of time (n = 24, 32.4%) and lack of training or awareness of guidelines (n = 23, 31.1%). Patient non-compliance (n = 14, 19.4%) and limited resources (n = 13, 17.6%) were reported less frequently.

Regarding educational needs, 68.9% of physicians (n = 51) indicated that they require additional training or resources to improve ankle sprain management. The majority of participants had not attended recent workshops or training sessions on musculoskeletal injury management (n = 65, 87.7%). Nevertheless, most physicians expressed willingness to receive further education on evidence-based ankle sprain management (n = 63, 85.1%) (Table 4).

**Table 4:** Training history and educational needs regarding ankle sprain management

Question	Response	Frequency (n)	Percent (%)
Need for additional training or resources	Yes	51	68.9
	No	23	31.1
Attended recent workshop/training	Yes	9	12.3
	No	65	87.7
Willingness to receive further education	Yes	63	85.1
	No	11	14.9

## Discussion

While the study population predominantly consisted of early-career physicians (defined as <5 years of clinical experience), our findings demonstrated no significant association between years of clinical experience and adherence to the OAR. However, this finding must be interpreted with caution; due to the very small number of participants in the higher-experience categories (n = 4 in both the 11-20 and >20 years groups), the study was likely underpowered to detect a true difference, posing a risk of a Type II error. Variability in OAR adherence was observed, which may have important clinical implications, including the potential for unnecessary diagnostic imaging, increased healthcare costs, and avoidable radiation exposure. Integrating clinical guidelines into ankle sprain management remains challenging, primarily due to their complexity and the need for consistent adherence [2]. Previous studies have consistently demonstrated a gap between physicians’ awareness of evidence-based guidelines and their actual implementation in clinical practice, particularly in urgent care settings [3,10,13]. Research indicates that while healthcare providers may report being knowledgeable, they frequently fail to adhere to recommended treatments [3]. To address these challenges, future interventions should be guided by established implementation frameworks, such as the Consolidated Framework for Implementation Research (CFIR), to systematically identify and overcome local barriers regarding awareness and resources.

In our study, a striking discrepancy was observed: while 95.9% of physicians reported familiarity with the OAR, only 56.8% correctly identified the primary criteria for tenderness at the posterior edge of the lateral malleolus. This highlights a significant gap between perceived familiarity and actual objective knowledge. Furthermore, while 81.1% of physicians recognized the benefits of early mobilization, only 41.9% routinely used structured rehabilitation protocols, directly highlighting a translation gap from knowledge to practice.

A substantial portion of physicians reported barriers, including time constraints (32.4%) and inadequate guideline training (31.1%). Notably, our data revealed a major access-to-CME (continuing medical education) problem: while 85.1% of physicians expressed a desire for additional education, 87.7% had not attended any recent training on musculoskeletal injury management. These findings suggest that educational initiatives alone may be insufficient unless accompanied by system-level changes that address structural and contextual challenges within urgent care environments [12,14].

Limitations

This study has several limitations. First, the cross-sectional design precludes causal conclusions. Second, a significant limitation regarding questionnaire validity is that the author-developed instrument, while content-validated by experts, did not undergo formal reliability testing. Third, there is a high risk of sampling bias due to the use of non-random, convenience sampling, and the potential for non-response bias, as the overall response rate was not reported. Furthermore, the small sample size, primarily of family medicine physicians, limits the generalizability of the findings to other clinical settings. Finally, the analysis of demographic associations was underpowered due to small subgroup sizes in the higher-experience categories, increasing the risk of a Type II error. Adherence to guidelines was based on self-reported practices rather than direct clinical observation or patient-level outcomes.

## Conclusions

In conclusion, this study highlights that while the majority of urgent care physicians demonstrate a high level of awareness regarding the OAR and evidence-based management principles, the consistent application of structured rehabilitation protocols remains suboptimal. Most respondents acknowledge the importance of early mobilization and patient education; however, barriers such as time constraints, insufficient training, and lack of resources hinder full implementation. To bridge the gap between knowledge and practice, healthcare institutions should prioritize continuous professional development and ensure the availability of necessary rehabilitation resources. Furthermore, implementing routine audits and feedback mechanisms can help evaluate adherence and identify specific areas for improvement, ultimately enhancing patient outcomes and reducing unnecessary imaging.

## Appendices

**Table 5:** Study questionnaire

Q#	Section	Question	Response options
1	Demographics	Age	☐ Under 30 ☐ 30-39 ☐ 40-49 ☐ ≥50
2	Demographics	Gender	☐ Male ☐ Female
3	Demographics	Years of clinical experience	☐ <5 ☐ 5-10 ☐ 11-20 ☐ >20
4	Demographics	Specialty	☐ Family medicine ☐ Emergency medicine ☐ Orthopedics ☐ Other
5	Demographics	Practice setting	☐ General hospital ☐ Primary health center ☐ PSMMC
6	Knowledge	Are you familiar with the Ottawa Ankle Rules (OAR)?	☐ Yes ☐ No
7	Knowledge	The Ottawa Ankle Rules recommend ankle X-rays if:	☐ Bone tenderness ☐ Inability to bear weight ☐ Both
8	Knowledge	Which guideline(s) do you use for managing ankle sprains?	☐ NICE ☐ AAFP ☐ Other
9	Knowledge	Do you know the benefits of early mobilization and rehabilitation for ankle sprains?	☐ Yes ☐ No
10	OAR application	Criteria for ordering an ankle X-ray based on OAR	☐ Lateral malleolus tenderness ☐ Medial malleolus tenderness ☐ Inability to bear weight ☐ Anterior swelling
11	OAR application	Which of the following excludes the use of OAR?	☐ Age < 18 ☐ Multiple trauma ☐ Severe swelling ☐ Malleolar pain
12	Clinical practice	How often do you apply the Ottawa Ankle Rules in practice?	☐ Always ☐ Often ☐ Sometimes ☐ Rarely ☐ Never
13	Clinical practice	Do you educate patients about injury prevention and rehabilitation?	☐ Yes ☐ No
14	Clinical practice	What treatment approach do you usually recommend? (Select all that apply)	☐ RICE ☐ NSAIDs ☐ Early mobilization ☐ Bracing/taping ☐ Physiotherapy
15	Clinical practice	Do you use structured rehabilitation protocols for ankle sprains?	☐ Yes ☐ No
16	Barriers	What barriers prevent adherence to evidence-based guidelines?	☐ Lack of time ☐ Lack of training ☐ Lack of resources ☐ Patient non-compliance
17	Education	Would you like further education on evidence-based ankle sprain management?	☐ Yes ☐ No
